# Acute Hematogenous Osteomyelitis in a Five-Month-Old Male with Rickets

**DOI:** 10.1155/2017/4627905

**Published:** 2017-10-31

**Authors:** Minnie John, Aseel Al-Jadiri, Christabelle Co, Maher Abulfaraj, Lucia J. Santiago

**Affiliations:** New York Presbyterian Brooklyn Methodist Hospital, Brooklyn, NY, USA

## Abstract

Osteomyelitis is defined as an infection of the bone, bone marrow, and the surrounding soft tissues. Most cases of acute hematogenous osteomyelitis in children are caused by Gram-positive bacteria, principally *Staphylococcus aureus*. We present a case where a 5-month-old male had an acute onset of decreased movement of his left leg and increased irritability and was subsequently diagnosed with rickets and hematogenous osteomyelitis with bacteremia. The case explores a possible association between hematogenous osteomyelitis and rickets.

## 1. Introduction

Osteomyelitis is defined as an infection of the bone, bone marrow, and the surrounding soft tissues [1]. The condition may occur for various reasons and can affect people of all ages. In childhood, the most common route of infection is via hematogenous spread of a microorganism [1]. Less common, osteomyelitis may also occur via contiguous spread such as after direct trauma.

In the pediatric population, boys are affected nearly twice as often as girls [1]. More than half of pediatric cases occur in children younger than five years, and one-quarter of cases occur in children younger than two years [2]. In infants less than 4 months old, osteomyelitis is uncommon without the presence of an underlying risk factor [3]. Risk factors include sickle cell disease, immunodeficiency, sepsis, minor trauma with coincident bacteremia, and indwelling vascular catheter [1]. Most cases of acute hematogenous osteomyelitis in children are caused by Gram-positive bacteria, principally *S. aureus* [1].

In this case report, we present a 5-month-old male who had an acute onset of decreased movement of his left leg and increased irritability and was subsequently diagnosed with rickets and hematogenous osteomyelitis with bacteremia. The patient had no other significant medical history and no risk factors associated with osteomyelitis. We aim to explore a connection between rickets and low bone density and vitamin D levels, as a potential risk factor for acquiring osteomyelitis. Furthermore, we speculate whether or not the coexisting conditions may affect the patient's treatment outcomes and prognosis, since vitamin D is extremely relevant in bone health [4].

## 2. Case Report

A five-month-old male, with no significant past medical history, was brought to the pediatric emergency department by his mother due to refusal to move his left lower limb and irritability for the past few hours prior to arrival. His mother denied any trauma or fever. The patient had no upper respiratory symptoms or recent illness. The patient was exclusively breastfed, and not on vitamin D supplementation. He was making adequate wet diapers, and there were no changes in bowel movements. The patient was born full term and delivered at home via normal spontaneous vaginal delivery. Newborn screening was negative with no evidence of sickle cell disease or immunodeficiencies that were routinely checked. The mother did not receive any prenatal care prior to delivery, and the patient's postnatal course was uneventful. As per the parent's wish, both the patient and his older sister were unimmunized.

**Figure 1:**
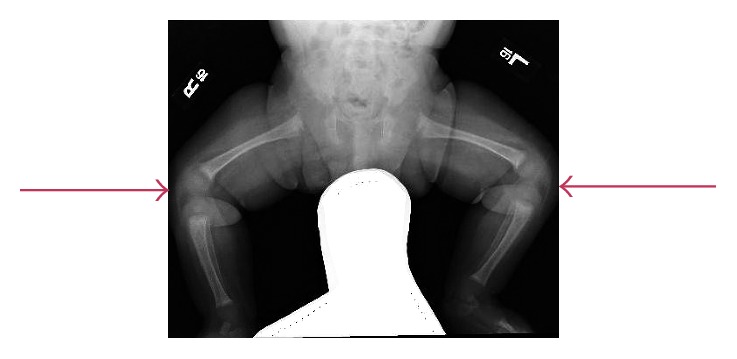
Osteopenia with fraying and cupping of the metaphysis suggestive of rickets.

Upon arrival to the emergency department, it was noted that the patient was irritable. He had a temperature of 101 Fahrenheit, and his left lower extremity was kept in the frog position. There was no erythema or swelling, but it was noted that he was crying upon palpating the extremity. Other than the left lower extremity findings, his physical exam was unremarkable. Given the findings on his physical exam, initial suspicion for infectious etiology versus child abuse was raised, and laboratory tests were sent including complete blood count with differential, inflammatory markers of C reactive protein (CRP) and erythrocyte sedimentation rate (ESR), blood culture, basic metabolic panel, and liver function tests. Results are as follows: white blood cell count of 9.9 K/uL; manual differential of 34% lymphocyte, 53% neutrophils, and 3% bands; hemoglobin level of 8.2 g/dL; very low level of calcium 5.9 mg/dL (normal range for age and sex 8.7–10.5 mg/dL); CRP of 22.8 mg/L; and ESR 30 mm/HR (Table 1). Imaging of the lower extremities was suggestive of rickets. No acute fracture was seen (Figure 1).

On the pediatric floor, the patient was started on acetaminophen every four hours, as well as calcium, vitamin D, and iron supplementation. The patient continued to be febrile with the highest temperature peaking to 104.4 F on day 2 of admission. Consults were obtained from the pediatric endocrine, orthopedic, and infectious disease specialists. A clinical decision was made to obtain further laboratory work including vitamin D, parathyroid hormone, phosphorus, and alkaline phosphatase levels to investigate the cause of hypocalcemia, which were significant for a vitamin D level of 9.2 ng/mL, parathyroid hormone level of 466 pg/mL, and normal phosphorus level of 3.1 mg/dL (Table 1). These labs were consistent with nutritional rickets, and the radiology findings were classical for rickets (Figure 1). The patient was started on vancomycin after microbiology informed us that the blood and urine cultures were both positive for Gram-positive cocci in clusters and to cover for Methicillin-resistant *Staphylococcus aureus*. The susceptibilities returned the next day, and the *Staphylococcus aureus* was methicillin sensitive (MSSA). Vancomycin was switched to cefazolin, and a repeat blood culture was immediately drawn, which was negative.

**Figure 2:**
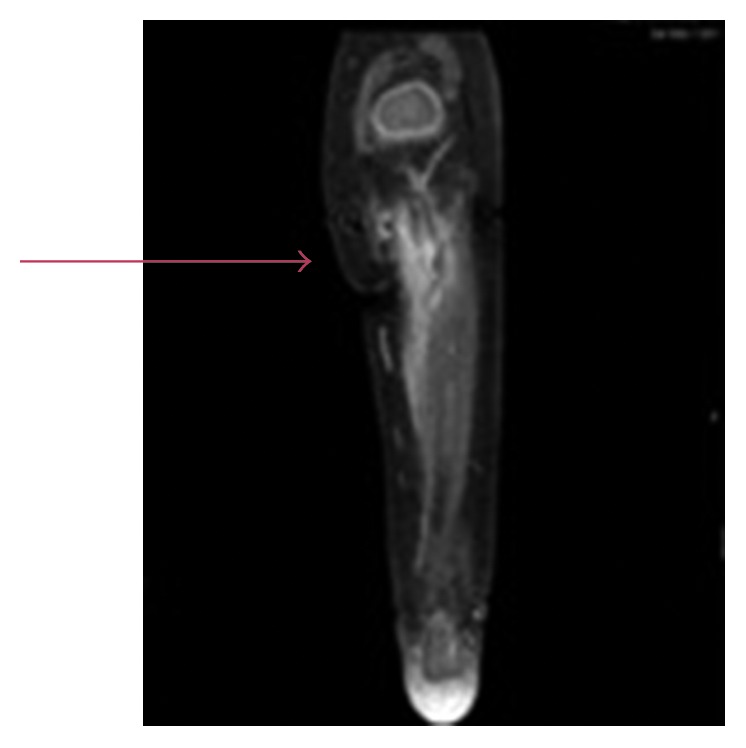
Minimal soft tissue edema surrounding the left proximal tibial metaphysis and diaphysis with corresponding mild enhancement suggestive of early acute osteomyelitis.

A magnetic resonance imaging (MRI) of the bilateral extremities was done on day 2 of admission which showed a small left knee effusion, possible myositis or cellulitis; however, it was noted to be a limited study. A repeat MRI was performed on day 6 of admission, which revealed osteomyelitis involving left proximal tibial diaphysis and metaphysis (Figure 2). On day 10 of admission, a peripherally inserted central catheter (PICC) was placed, and antibiotic was switched to nafcillin every 6 hours. Nafcillin causes increased rates of phlebitis in children with peripheral IVs, and hence cefazolin was preferred when the child had a peripheral IV. Therefore, after placing the PICC, the antibiotic was changed from cefazolin to nafcillin, as nafcillin is the drug of choice for MSSA. We switched the antibiotic to nafcillin from cefazolin, because nafcillin could cause phlebitis if given through a peripheral IV in a child, has a narrow spectrum, and is the drug of choice for MSSA.

He was treated with 16 days of antibiotics as inpatient, and his inflammatory markers (ESR and CRP) and calcium and phosphorus were trended every 2–3 days and were normal at the time of discharge.

He was discharged home with vitamin D supplements and a 4-week-course of oral clindamycin. The patient was followed up three times with the infectious disease and endocrinology clinics. Repeat Ca and phosphorus levels after discharge remained normal with an improved vitamin D level of 16.9 ng/mL. The vitamin D lab was never redone since patient was lost to follow-up after 32 days post discharge.

## 3. Discussion

Our case is unique in that the patient was diagnosed with vitamin D deficiency, rickets, and osteomyelitis, all conditions that are well known to occur amongst the pediatric population; however, they are not commonly found together. To our knowledge, this is one of the first reported cases in pediatrics. Other cases report hematogenous osteomyelitis caused by the presence of bacteria or vitamin D deficiency; however, there are no documented case repots illustrating osteomyelitis and the diagnosis of rickets [1]. We hope to provide further insight into a possible association and/or correlation between the pathologies.

Rickets is a common preventable disease that leads to the under mineralization of bones and subsequently multiple bone deformities. The most common cause of rickets is due to nutritional vitamin D deficiency. We suspect that this was the case in our patient, as he was exclusively breastfed and did not receive any vitamin supplementation. Another risk factor that may have contributed is that he was born during the winter months with limited sun exposure.

The diagnosis of nutritional rickets is made via history and physical, biochemical testing, and radiographs. A vitamin D (25-OHD) level of less than 30 nmol/L is considered deficient [2, 5]. A decrease in serum phosphorus and calcium is also suspected. Reversely, serum PTH, ALP, and urinary phosphorus levels are increased. Due to defective mineralization, growth plate widening and metaphyseal cupping and fraying can be seen on X-ray which confirm the diagnosis of rickets. Treatment for nutritional rickets is vitamin D supplementation of at least 2000 IU daily for three months, after which a 25-OHD concentration should be repeated to determine whether the supplementation should be continued [5].

Osteomyelitis is a devastating bone infection that affects children with a median age of 6 years. Patients present with an acute history of bone pain, often the long bones of the lower limbs. Signs of inflammation such as redness, warmth, and swelling do not appear unless the infection has progressed deep into the bone. Risk factors for hematogenous osteomyelitis amongst older infants and children include sickle cell disease, immunodeficiency disorders, sepsis, minor trauma coincident with bacteremia, and indwelling vascular catheters. Our child did not have any risk factors including immunodeficiencies and sickle cell disease. Due to the combination of fever with pain in his lower extremity, osteomyelitis was clinically suspected as a differential and was later confirmed by MRI.

Osteomyelitis varies geographically with it being less common in developed countries, 1 in 5000–7700 children, as opposed to 1 in 500–2300 children in developing countries [1]. *Staphylococcus aureus* is the most common cause in children [1]. The United States has reported an increase in the incidence over time due to the emergence of community-associated methicillin-resistant *Staphylococcus aureus*. Bacteria deposit in the metaphysis of long bones and lead to cellulitis in the bone marrow. Osteomyelitis requires antimicrobial therapy, initially parenteral. After the patient shows clinical signs of improvement and the ESR or CRP decreases, he can be switched to oral therapy for a total of four–six weeks regimen. Recent studies have shown that shorter courses of antibiotics are equally effective [6]. We are aware that the newer guidelines recommend shorter courses of treatment; however, there are no reported cases of osteomyelitis and rickets, and we elected to do the longer course in this case as we were not sure of the healing process in a bone with osteomyelitis. Empiric antibiotics are first given and should be tailored to a specific pathogen when culture and susceptibility results are available.

Vitamin D and calcium are both well known to play an important role in bone development and strength. Furthermore, vitamin D has been increasingly recognized as an immune mediator that may play a role in the pathogenesis of infections [7]. A study in Europe was done which reviewed observational cohort studies on vitamin D deficiency in the intensive care unit between 2000 and 2014. It showed that vitamin D deficiency (<50 nmol/L) is associated with an increase in infection rate, sepsis, 30-day mortality, and inhospital mortality in adult critically ill patients, worldwide [4]. After a thorough literature search, only a few articles have been published regarding a possible connection between low calcium and vitamin D deficiency with the development of bone infections [7, 8]. These studies showed that there was an increased prevalence in vitamin D deficiency among those patients who concurrently had a bone infection, but not in pediatric population specifically. An exact causal relationship could not be established, but it was suggested that vitamin D supplementation may be a possible way to lower the risk of periprosthetic joint infection [7, 8].

## 4. Conclusion

In summary, we highlight that since infants already have a thinner bone cortex, they are more prone to hematogenous spread of infection into the bone. The addition of vitamin D deficiency and hypocalcemia may further put them at an even higher risk for osteomyelitis. To our knowledge, this is the first documented pediatric case that documents a possible link between vitamin D deficiency, rickets, and osteomyelitis. It is currently unknown whether vitamin D has a specific role in the bone and joint infections. It is our hope that, with this case, future cases will be reported and the association between the diagnoses can be further explored.

## Figures and Tables

**Table 1:** Lab results during hospital course and post discharge.

	Admission Days				
Labs (normal range)	Day 1 4/13	Day 7 4/20	Day 12 4/25	Day 16 discharge 4/29	Day 26 post discharge
WBC (6.0–17.5 K/uL)	9.9 K/uL	12.8 K/uL	7.9 K/uL	8.3	7.9 K/uL
Alkaline (53–1218 Unit/L)	2,121 Unit/L	1,774 Unit/L	—	1,862	—
ESR (0–15 MM/HR)	30 MM/HR	—	29 MM/HR	14 MM/HR	12 MM/HR
CRP (<3.0 mg/L)	22.8 mg/L	<2.9 mg/L	—	—	—
Calcium (8.5–10.1 mg/dL)	5.9 mg/dL	9.6 mg/dL	9.7 mg/dL	9.3 mg/dL	—
Vitamin D (30–100 ng/mL)	9.2 ng/mL	—	—	16.5 ng/mL	—
Parathyroid (14–72 pg/mL)	466 pg/mL	—	—	15 pg/mL	—
